# Near-Infrared Spectroscopy, a Rapid Method for Predicting the Age of Male and Female Wild-Type and *Wolbachia* Infected *Aedes aegypti*

**DOI:** 10.1371/journal.pntd.0005040

**Published:** 2016-10-21

**Authors:** Maggy T. Sikulu-Lord, Masabho P. Milali, Michael Henry, Robert A. Wirtz, Leon E. Hugo, Floyd E. Dowell, Gregor J. Devine

**Affiliations:** 1 Mosquito Control Laboratory, QIMR Berghofer Medical Research Institute, Brisbane, Queensland, Australia; 2 Environmental Health and Ecological Sciences Thematic Group, Ifakara Health Institute, Ifakara, United Republic of Tanzania; 3 Centers for Disease Control and Prevention, Atlanta, Georgia, United States of America; 4 Stored Product Insect and Engineering Research Unit, Center for Grain and Animal Health Research, Agricultural Research Service, United States Department of Agriculture, Manhattan, Kansas, United States of America; Centers for Disease Control and Prevention, Puerto Rico, UNITED STATES

## Abstract

Estimating the age distribution of mosquito populations is crucial for assessing their capacity to transmit disease and for evaluating the efficacy of available vector control programs. This study reports on the capacity of the near-infrared spectroscopy (NIRS) technique to rapidly predict the ages of the principal dengue and Zika vector, *Aedes aegypti*. The age of wild-type males and females, and males and females infected with *w*Mel and *w*MelPop strains of *Wolbachia pipientis* were characterized using this method. Calibrations were developed using spectra collected from their heads and thoraces using partial least squares (PLS) regression. A highly significant correlation was found between the true and predicted ages of mosquitoes. The coefficients of determination for wild-type females and males across all age groups were R^2^ = 0.84 and 0.78, respectively. The coefficients of determination for the age of *w*Mel and *w*MelPop infected females were 0.71 and 0.80, respectively (*P*< 0.001 in both instances). The age of wild-type female *Ae*. *aegypti* could be identified as < or ≥ 8 days old with an accuracy of 91% (N = 501), whereas female *Ae*. *aegypti* infected with *w*Mel and *w*MelPop were differentiated into the two age groups with an accuracy of 83% (N = 284) and 78% (N = 229), respectively. Our results also indicate NIRS can distinguish between young and old male wild-type, *w*Mel and *w*MelPop infected *Ae*. *aegypti* with accuracies of 87% (N = 253), 83% (N = 277) and 78% (N = 234), respectively. We have demonstrated the potential of NIRS as a predictor of the age of female and male wild-type and *Wolbachia* infected *Ae*. *aegypti* mosquitoes under laboratory conditions. After field validation, the tool has the potential to offer a cheap and rapid alternative for surveillance of dengue and Zika vector control programs.

## Author Summary

*Aedes aegypti* is the principal vector for dengue, chikungunya and Zika viruses. These viruses require a period of development inside the mosquito before they can be transmitted to humans. Depending on environmental factors, dengue and Zika viruses take an average of 8–10 days to replicate inside the mosquito [1,2] whereas chikungunya virus takes 2–7 days to replicate [3]. The age of mosquitoes is therefore a critical determinant of disease transmission. A mosquito control strategy utilizing an endosymbiotic bacterium *Wolbachia pipientis*, has been proven effective at blocking dengue transmission in *Ae*. *aegypti* mosquitoes [4]. For effective virus blocking, mosquitoes infected with the *w*Mel strain of *Wolbachia* must survive long enough to cover the extrinsic incubation period (EIP) of infected mosquitoes while those infected with *w*MelPop are expected to have a reduced lifespan to limit the period available for virus replication. These strategies therefore require routine monitoring using age grading techniques. In this study, we investigated the applicability of a rapid and cost effective near-infrared spectroscopy (NIRS) technique as an alternative age grading tool for wild-type and *Wolbachia* infected *Ae*. *aegypti* mosquitoes. We show that NIRS can rapidly predict on average, the age of male and female *Ae*. *aegypti* to ±3 days of their true age and can determine which mosquitoes are old enough to be potentially infectious with an accuracy of up to 91%.

## Introduction

The mosquito *Aedes aegypti* is the primary vector for dengue, Zika and chikungunya viruses. Up to 100 million dengue cases occur annually [5,6] and an estimated 440,000–1,300,000 Zika cases were reported in early 2016. Notably, 3893 babies born to Zika infected mothers have been diagnosed with microcephaly [7,8]. Both Zika and dengue viruses are transmitted by female *Ae*. *aegypti* mosquitoes carrying viruses in their salivary glands. Due to the period required by the virus to replicate inside the mosquito, *Ae*. *aegypti* mosquitoes are in most cases only capable of transmitting the Zika or dengue viruses when they are at least 8 days old [1,2,9].

The success of existing arbovirus vector control programs for mosquito-borne viruses recommended by the World Health Organization such as targeted residual spraying and space spraying is dependent on their ability to shorten the lifespan of the mosquito and to limit the period available for virus development. Alternative biological control strategies under trial involve the release of *Ae*. *aegypti* mosquitoes transinfected with the *w*Mel strain of *Wolbachia pipientis* for the suppression of arbovirus transmission [4] or the pathogenic *w*MelPop strain for arbovirus and/or vector population suppression [10–12]. Although the efficacy of *Wolbachia* induced arbovirus interference was first demonstrated against dengue and Chikungunya [13], it has now also been demonstrated against Zika virus [14,15]. Unlike the life-shortening strain *w*MelPop, the success of *w*Mel as a biological control agent is dependent upon the survival of infected mosquitoes [4].

Following the outbreak of Zika virus in South America in 2015, there is renewed interest in defining mosquito survival characteristics as a means of evaluating vector control strategies that affect adult mosquito survival. The efficacy of the WHO recommended control interventions [16], would therefore be defined by their ability to effectively eliminate the old and potentially infectious population. To accurately define these characteristics, evaluations of current interventions would require assessments of mosquito populations on large-scale using rapid and accurate age grading techniques.

Traditionally, entomologists have used techniques based on dissections of the female reproductive system to estimate mosquito age and survival. These include the Detinova technique for differentiating parous and nulliparous mosquitoes [17] and the Polovodova technique for determining the number of times mosquitoes have laid eggs [18]. However, these techniques are time consuming and labour-intensive. Moreover, interpretation of the diagnostic changes to ovarian morphology can be problematic [19]. As a result, estimates of population age structure based on these techniques often lack statistical power as only a small sample size can be dissected within a reasonable timeframe.

Analysis of age-related changes of cuticular hydrocarbons by gas chromatography [20–22] and transcriptional profiling [23] have been evaluated as alternative age grading techniques for *Aedes* mosquitoes. However, their high costs may restrict their utility and sustainability for large-scale field trials or control programs, particularly in areas where resources are limited. Although age related proteomic changes recently reported for *Ae*. *aegypti* [24] may offer cost effective and rapid alternative means of age assessments, these techniques are still early in development.

New approaches are required that can rapidly and cost-effectively assess large numbers of field specimens. In our previous studies, we reported the use of the near-infrared spectroscopy (NIRS) technique for age and species prediction. The tool was first applied to predict the age of female laboratory reared *An*. *gambiae* and *An*. *arabiensis* and to differentiate these sibling species [25]. It was then used to age grade and differentiate species of semi field reared *An*. *gambiae* and *An*. *arabiensis* [26], age grade laboratory reared *An*. *arabiensis* undergoing various physiological changes [27], preserved specimens [28–30] and to age grade *Anopheles gambiae* sensu lato mosquitoes reared from wild larvae that were either susceptible or resistant to pyrethroids [31]. More recently NIRS successfully predicted the ages of laboratory *Ae*. *aegypti* maintained on a varying larval and adult diets [32]. From laboratory and semi-field studies, we have shown that the accuracy of NIRS for *An*. *gambiae* s.s. and *An*. *arabiensis* ranges between 79–90% and 80–90% for age grading into <7 d and ≥ 7 d old age groups and for species differentiation, respectively.

Given its rapidity and relative ease of application, NIRS represents a unique opportunity to develop a viable alternative to current ovarian dissections and molecular techniques for age grading. The use of a NIR spectrometer for this purpose is non-destructive, rapid, and requires little training. NIRS facilitates the analysis of hundreds of mosquitoes just in one day because it takes 15 seconds to prepare and collect spectra from a mosquito, without reagents or sample preparation procedures. Moreover, samples can be scanned either fresh or preserved [28–30].

Here, we show that NIRS may be used to predict the age of both male and female wild-type *Ae*. *aegypti* mosquitoes up to 30 d old. We also examined the ability of NIRS to predict the age of *Ae*. *aegypti* females and males infected with *w*Mel and *w*MelPop strains of *Wolbachia pipientis*.

## Methods

### Ethics approval

Ethics approvals were obtained for routine blood feeding of mosquito colonies from the QIMR Berghofer Medical Research Institute (QIMR HREC P1162). Written informed consent was provided by all adult volunteers involved in blood feeding, and volunteers were given the right to refuse to participate or withdraw from the experiment at any time.

### Mosquito rearing

Colonies of wild-type *Ae*. *aegypti* and *Ae*. *aegypti* infected with *w*Mel and *w*MelPop were acquired and maintained at the insectary of QIMR Berghofer Medical Research Institute as previously described [33]. Adults were given a 24 hr window to emerge. Wild-type females and males were collected at 1, 5, 9, 13, 17, 21, 25 and 30 d post emergence. Adult *w*Mel strain mosquitoes were collected at 1, 5, 10, 15, 19 and 20 d post emergence. Adult *w*MelPop strain mosquitoes were collected at 1, 5, 10, 15 and 19 d post emergence. All mosquitoes were collected either unfed (1 and 5 d old) or blood fed but after oviposition (> 5 days old mosquitoes). Adults were knocked down with carbon dioxide and stored in RNA*later* solution. To allow for RNA*later* penetration, samples were stored overnight in a 4°C refrigerator and then stored at -20°C for 2 months before scanning [28].

### Mosquito scanning using NIR spectrometer

**Fig 1:**
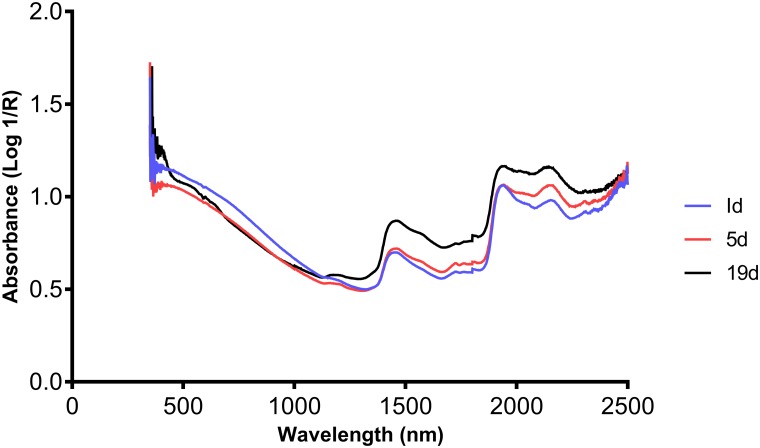
Example of typical raw spectra collected from heads and thoraces of *w*Mel infected female *Ae*. *aegypti* at 3 different age points.

All mosquitoes collected were transferred to Ifakara Health Institute, Tanzania for scanning. Prior to scanning, residual RNA*later* was removed from the mosquito specimens by blotting with paper towels. A maximum of 25 mosquitoes at a time were then positioned on a spectralon plate (ASD Inc, Boulder, CO), ventral side up. At least 40 mosquitoes at each age for all species were scanned using a LabSpec 5000 NIR spectrometer (ASD Inc, Boulder, CO), according to previously described protocols [14]. To collect the spectra, the heads and thoraces of mosquitoes were scanned under a 3 mm-bifurcated fibre optic probe containing six collection fibres and 33 illumination fibres. Typical raw spectra collected from the head and thorax of female *w*Mel infected *Ae*. *aegypti* at 1, 5 and 19 d age points are shown in Fig 1.

## Data analysis

Spectra were analyzed using Grams IQ software (Thermo Galactic, Salem, NH). Due to low light energy at shorter wavelengths and low sensor sensitivity at longer wavelengths, spectra appeared noisy outside the 500–2350 nm region. Therefore results were analyzed using spectra measured within this region. The relationship between spectra and age were analyzed by partial least squares (PLS) regression.

**Fig 2:**
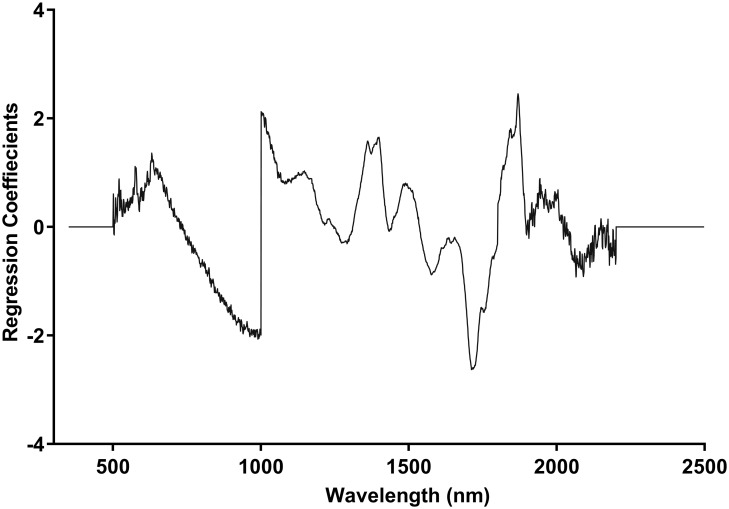
Regression coefficients for predicting the age of female wild-type *Ae*. *aegypti* using 9 partial least squares regression factors.

For wild-type *Ae*. *aegypti*, mosquitoes were divided into a training set and a validation set. The training set is used for developing a calibration model using cross validation analysis. Cross-validation is a “leave-one-out” self-prediction method where mosquitoes from a set are used to predict the age of mosquitoes from that same set. It is used to select the factors required for the calibration of the predictive model before running the validation set. All *Wolbachia* infected mosquitoes were analyzed using the cross validation method. The number of factors used in developing models was determined from the cross-validation prediction residual error sum of squares (PRESS) and regression coefficient plots. An example of a coefficient plot used for predicting the age of female wild-type *Ae*. *aegypti* is shown in Fig 2. Analysis of variance (ANOVA) was applied to test for statistical differences between mean predicted ages using the Statistical Package for Social Sciences 22 (IBM, Armonk, NY). Tukey post hoc analysis in ANOVA was applied to test for statistical differences between age groups. Actual age and predicted age were coded as independent and dependent variables, respectively. The relationship between true and predicted age was assessed by Spearman’s rank correlation coefficient analysis.

## Results

### Age prediction of male and female wild-type *Ae*. *aegypti*

**Fig 3:**
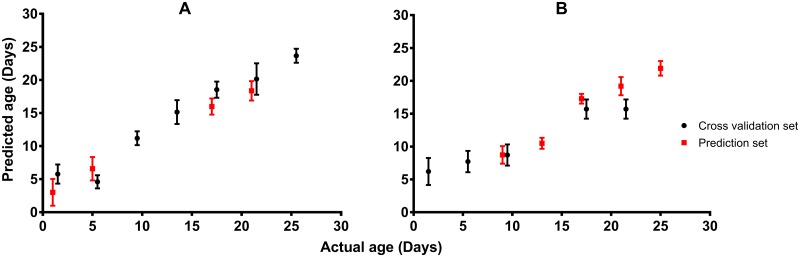
Mean (95% CI) age prediction of female (A) and male (B) of wild-type *Ae*. *aegypti* mosquitoes using NIRS.

Except for 1 d old mosquitoes, the mean predicted age of female *Ae*. *aegypti* mosquitoes was within ± 2d of the actual age across all age groups (Table 1; Fig 3A). An accuracy of 91% (N = 501) was achieved if female mosquitoes excluded from the model were simply grouped into two age categories that separate mosquitoes that are unlikely to be infectious (< 8 d) from those that are old enough to have survived the dengue/Zika incubation period and therefore would be potentially infectious (≥ 8 d). Additionally, Spearman’s correlation analysis indicated a strong positive correlation between the predicted and the actual age for both the training set (R^2^ = 0.84; *P* <0.001) and the validation set (R^2^ = 0.83; *P* <0.001). Tukey Post hoc analysis indicated that female *Ae*. *aegypti* could be differentiated into 5 age groups (1–5, 6–9, 10–13, 14–17 and >17 d old).

**Table 1:** Mean age predictions of female and male *wild-type Ae*. *aegypti* mosquitoes using the cross validation method for samples used in the model and the prediction method for samples that were excluded from the model.

Wild-type *Ae*. *aegypti* females						Wild-type *Ae*. *aegypti* males					
	Cross validation set^1^ [N = 101]		Prediction set^2^ [N = 501]			Cross validation set^1^ [N = 142]			Prediction set^2^ [N = 253]		
Actual age	Mean predicted age [95% CI]	SEM	Actual age	Mean predicted age [95% CI]	SEM	Actual age	Mean predicted age [95% CI]	SEM	Actual age	Mean predicted age [95% CI]	SEM
1	3.0^a^[0.9–5.6]	0.9	1	5.3^a^ [3.8–6.7]	0.7	1	5.7^a^[3.6–7.7]	0.9	9	8.5^a^ [7.1–10.0]	0.7
5	6.5^b^[4.8–8.3]	0.8	5	4.1^a^ [3.1–5.1]	0.5	5	7.2^a,b^[5.5–8.8]	0.7	13	10.4^a^ [9.5–11.4]	0.5
17	15.9^c^[14.7–17.2]	0.5	9	10.7^b^ [9.6–11.7]	0.5	9	8.2^a,b^[6.6–9.8]	0.7	17	16.8^b^ [15.9–17.8]	0.5
21	18.3^c^[16.8–19.8]	0.7	13	14.6^c^ [12.8–16.4]	0.9	17	15.2^c^[13.7–16.6]	0.7	21	19.5^c^ [18.3–20.8]	0.6
			17	18.0^d,f^ [16.8–19.3]	0.6	21	17.3^c^[15.9–18.7]	0.6	25	21.5^c^ [20.5–22.6]	0.5
			21	19.6^c,d,e,f^ [17.3–22.0]	0.9	30	28.1^d^[24.8–31.8]	1.6	30		
			25	23.2^e^ [22.1–24.2]	0.5						
			30	19.9^f^[18.9–20.9]	0.3						

Means followed by the same letter are not significantly different at *P*<0.05 when using Tukey post hoc test

Actual and mean predicted ages shown are in days

^1^ The accuracy of samples used to develop calibration models

^2^ The accuracy of samples used to validate models

The age prediction of male mosquitoes that were excluded from the training set was generally within ± 3 d of the actual age. Moreover, an overall accuracy of 87% (N = 253) into < 8 d and ≥ 8 d old age groups was achieved. Spearman correlation analysis found a highly positive correlation between the predicted age and the actual age for both the training set (R^2^ = 0.77; *P* <0.001) and the validation set (R^2^ = 0.78; *P* <0.001). Tukey post hoc comparison test between age groups of male *Ae*. *aegypti* indicated that mosquitoes could be differentiated into four age groups (<9, 9–13, 14–17 and >17 d old) (Table 1; Fig 3B).

### Age prediction of males and females infected with *w*Mel and *w*MelPop

Using the cross validation analysis, females (N = 284) and males (N = 277) infected with the *w*Mel strain were differentiated into < 8 d and ≥ 8 d old age groups with accuracies of 83%. Males (N = 234) and females (N = 229) infected with the *w*MelPop strain were both differentiated into the two groups with an accuracy of 78%. Spearman correlation analysis found a positive correlation between the predicted age and the actual age of females (R^2^ = 0.71; *P*<0.001) and males (R^2^ = 0.80; *P*<0.001) infected with *w*Mel and females (R^2^ = 0.80; *P*<0.001) and males (R^2^ = 0.68; *P*<0.001) infected with *w*MelPop. All *Wolbachia* infected females and males were generally differentiated into 4 age groups (<5, 5–9, 10–15 and >15 d old) (Table 2).

**Table 2:** Mean age predictions of female and male *w*Mel and *w*MelPop infected *Ae*. *aegypti* mosquitoes using the cross validation method.

*w*Mel infected *Ae*. *aegypti*: Cross Validation						*w*MelPop infected *Ae*. *aegypti*: Cross validation					
	*w*Mel infected females [N = 284]		*w*Mel infected males [N = 277]			*w*MelPop infected females [N = 229]			*w*MelPop infected males [N = 234]		
Actual age	Mean predicted age [95% CI]	SEM	Actual age	Mean predicted age [95% CI]	SEM	Actual age	Mean predicted age [95% CI]	SEM	Actual age	Mean predicted age [95% CI]	SEM
1	2.8^a^[1.6–3.9]	0.5	1	4.8^a^[3.9–5.6]	0.4	1	2.4^a^[1.4–3.9]	0.5	1	3.9^a^[2.9–5.0]	0.5
5	10.0^b^[9.2–10.7]	0.3	5	6.4^ab^[5.6–7.2]	0.3	5	9.0^b^[8.3–9.8]	0.3	5	8.9^b^[7.9–9.8]	0.4
10	13.2^c^[12.3–14.1]	0.4	10	7.6^b^[6.6–8.6]	0.4	10	9.7^b^[8.6–10.8]	0.5	10	11.0^c^[10.1–11.9]	0.4
15	13.6^c^[12.4–14.7]	0.5	15	15.8^c^[15.1–16.5]	0.3	15	12.6^c^[11.6–13.6]	0.4	15	12.2^c^[11.0–13.4]	0.5
19	14.6^c,d^[13.6–15.7]	0.5	19	16.7^c^[15.4–18.1]	0.6	19	16.4^d^[15.6–17.2]	0.4	19	14.3^d^[13.3–15.2]	0.4
20	15.8^d^[14.9–16.7]		20	16.7^c^[15.9–17.6]	0.4						

Means followed by the same letter are not significantly different at *P*<0.05 when using Tukey post hoc test

Actual and mean predicted ages shown are in days

^1^ The accuracy of samples used to develop calibration models

## Discussion

Our findings demonstrate the potential of the NIRS as a rapid technique for identifying the age of wild-type and *Wolbachia* infected *Ae*. *aegypti* mosquitoes. Mosquito survival is a fundamental parameter of vectorial capacity. As 1–2 days is required for blood feeding and at least 7 days is required for virus replication [2,9,34], the average infectious age of *Ae*. *aegypti*, the principal vector for Zika and dengue viruses, is considered to be at least 8 days. *Wolbachia*-based strategies utilizing the *w*Mel strain require infected mosquitoes to survive and mate effectively with wild mosquitoes to drive the bacteria through populations. Alternatively, the life shortening properties of the pathogenic *w*MelPop strain may be harnessed to crash local vector populations [11]. The validation of either strategy will require accurate mosquito age grading techniques that can function against a background of *Wolbachia* infection.

Recently, Liebman and colleagues used NIRS to predict the age of laboratory reared female *Ae*. *aegypti* mosquitoes maintained on varying larval and adult diet up to 16 days post emergence [32]. We report on the ability of NIRS to age grade female and male wild-type *Ae*. *aegypti* up to 30 d old as well as females and males *Ae*. *aegypti* infected with *Wolbachia* up to 20 d old. Age predictions were determined on a continuous age scale and into < 8 d or ≥ 8 d old age groups. Although shorter EIPs have been reported for dengue viruses [35], eight days was the favored cut off point because it is widely quoted as the average age at which *Ae*. *aegypti* or *Ae*. *albopictus* are able to transmit dengue or Zika viruses [2,9,34] and the best accuracy was achieved at this cut off point.

Wavelengths ranging from 500–2350 nm were analyzed. This range comprises carbon-hydrogen (C-H) functional groups at 1220, 1450, 1700 and 1765 nm and protein (N-H) functional groups at 1510, 2055, 2060, 2180 and 2300 nm. Both cuticular hydrocarbons [20,21] and proteins [24] have previously yielded biomarkers suitable for age grading *Ae*. *aegypti* mosquito species. Overall, age prediction of wild-type females and *Wolbachia* infected females across all age categories assessed was within ±3–5 days of actual age. Five day old females infected with *Wolbachia* were the least accurately predicted. It may be that few developmental changes occur between 5 and10 days. It was recently reported by Hugo and colleagues, who examined changes in protein abundance with age of *Ae*. *aegypti*, that age-related changes for the majority of the proteins reported occurred between 1 and 5 d with little or no further protein changes occurring among older age groups [24]. These proteomic and transcriptome approaches may ultimately help identify biomarkers contributing to age related variation in NIRS spectra, provide further validation and improve the prediction accuracy of NIRS. We found strong positive correlation between the actual age and the predicted age for both wild-type and *Wolbachia* infected mosquitoes.

Age prediction accuracy of male wild-type and male mosquitoes infected with *Wolbachia* was impressive. NIRS predicted the ages of male wild-type, *w*Mel and *w*MelPop mosquitoes into < 8 and ≥ 8 d age groups with 87%, 83% and 78% accuracy, respectively. This is the first investigation to report the ability of NIRS to predict the age of male *Ae*. *aegypti* mosquitoes. Despite the fact that they are not disease vectors, the survival of male mosquitoes would be a useful indicator of the success or failure of control strategies utilizing *Wolbachia* [10], genetic modification approaches requiring male competitiveness [36,37], a strategy to induce sex-ratio distortion of mosquito populations towards males [38] and strategies that seek to release sterile males [39]. The efficacy of any sterile male technique, including those that utilise *Wolbachia* infected males, relies on the ability of the male mosquito to compete and mate with the wild-type population. Nonetheless, to date only one age grading technique has been reported for male mosquitoes. A technique based on the frequency at which spermatocysts are found in male reproductive organs of *An*. *gambiae* s.s. and *Anopheles culicifacies* and the relative size of the sperm reservoir to differentiate mosquitoes into ≤ 4 d old and > 4 d old [40,41]. The difficult dissections and complex quantitative models involved in the application of this age grading technique, as pointed out by the authors, may limit its application on field related studies. Given its rapidity and simple prediction models, NIRS would be a suitable complementary age grading tool for rapidly differentiating young from old male mosquitoes.

NIRS offers a considerably higher throughput than alternative mosquito age grading approaches. We estimate that we can analyze approximately 1000 mosquitoes per day. Comparatively, the analysis of samples by cuticular hydrocarbon or transcriptional profiling age grading techniques currently allows an average of only 20–30 samples to be processed per day (averaged over preparation periods) [42]. With the capacity for larger sample sizes to be processed, NIRS has the potential to reduce sampling error associated with age prediction estimates in comparison to the alternative age grading techniques. The increased capacity and reduced sampling bias has the potential for substantially increasing the accuracy of population survivorship estimates, provided age prediction models are constructed without bias [22].

Accurate determination of mosquito population survivorship is key to understanding transmission risk and to the success of vector control strategies. Until recently, it has been impractical or impossible to accurately assess the age of *Ae*. *aegypti* in the field. Traditional dissection methods are technically challenging and only accurately differentiate parous from non-parous mosquitoes. Excitingly, our results that NIRS can accurately age *Ae*. *aegypti* mosquitoes over a 3 week lifespan gives us a tool for assessing transmission risk and understanding seasonality. NIRS has considerable advantages over conventional techniques given that results can be gained in real-time, without reagents or sample preparation. To improve the accuracy, alternative statistical methods are being developed. However, as certain age groups may be biochemically indistinguishable from each other and since NIRS relies on assessing alterations in biochemical signatures, it should be acknowledged that shortcomings in age prediction may stem from the underlying biochemistry as opposed to the statistical techniques applied. Although our study is still preliminary, our results demonstrate that upon further calibration, NIRS could be a potentially more accurate tool for predicting the age of *Ae*. *aegypti* compared to transcriptional profiling and cuticular hydrocarbon techniques. Follow up studies conducted under semi-field or field environments to improve the current calibration models have been envisaged. These and our recently published findings that NIRS can detect *Wolbachia* infections in *Ae*. *aegypti* [33], suggest a strong potential role for NIRS as a rapid surveillance tool for *Ae*. *aegypti* control programs.
